# Bis(acrylonitrile-κ*N*)dichlorido(η^4^-cyclo­octa-1,5-diene)ruthenium(II)

**DOI:** 10.1107/S1600536811035380

**Published:** 2011-09-14

**Authors:** Haleden Chiririwa, Reinout Meijboom

**Affiliations:** a Research Centre for Synthesis and Catalysis, Department of Chemistry, University of Johannesburg, PO Box 524, Auckland Park, 2006 Johannesburg, South Africa

## Abstract

In the title complex, [RuCl_2_(C_8_H_12_)(C_3_H_3_N)_2_], the metal ion is coordinated to centers of each of the double bonds of the cyclo­octa-1,5-diene ligand, to two chloride ions (in *cis* positions) and to two N-atom donors from two acrylonitrile mol­ecules that complete the coordination sphere for the neutral complex. The coordination about the Ru^II^ atom can thus be considered octa­hedral with slight trigonal distortion. The three C atoms of one of the acrylonitrile ligands are disordered over two sets of sites in a 0.581 (13):0.419 (13) ratio.

## Related literature

For a review of related compounds, see: Chiririwa *et al.* (2011 ▶). For the synthesis of starting materials, see: Ashworth *et al.* (1987 ▶)
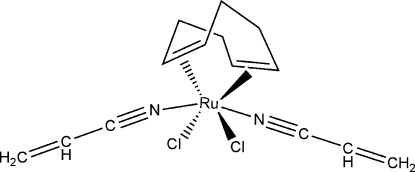


## Experimental

### 

#### Crystal data

[RuCl_2_(C_8_H_12_)(C_3_H_3_N)_2_]
*M* *_r_* = 386.27
Monoclinic, 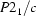

*a* = 7.1079 (8) Å
*b* = 26.818 (3) Å
*c* = 8.1555 (10) Å
β = 101.408 (2)°
*V* = 1523.9 (3) Å^3^
*Z* = 4
Mo *K*α radiation
μ = 1.37 mm^−1^
*T* = 100 K
0.22 × 0.09 × 0.04 mm

#### Data collection

Bruker APEXII CCD diffractometer
12653 measured reflections
3776 independent reflections
3093 reflections with *I* > 2σ(*I*)
*R*_int_ = 0.040

#### Refinement

*R*[*F* ^2^ > 2σ(*F* ^2^)] = 0.033
*wR*(*F* ^2^) = 0.075
*S* = 1.03
3776 reflections
200 parameters
H-atom parameters constrained
Δρ_max_ = 1.14 e Å^−3^
Δρ_min_ = −1.11 e Å^−3^

### 

Data collection: *APEX2* (Bruker, 2007 ▶); cell refinement: *SAINT-Plus* (Bruker, 2007 ▶); data reduction: *SAINT-Plus* and *XPREP* (Bruker, 2007 ▶); program(s) used to solve structure: *SHELXS97* (Sheldrick, 2008 ▶); program(s) used to refine structure: *SHELXL97* (Sheldrick, 2008 ▶); molecular graphics: *DIAMOND* (Brandenburg & Putz, 2005 ▶) and *ORTEP-3* (Farrugia, 1997 ▶); software used to prepare material for publication: *WinGX* (Farrugia, 1999 ▶).

## Supplementary Material

Crystal structure: contains datablock(s) global, I. DOI: 10.1107/S1600536811035380/jh2321sup1.cif

Structure factors: contains datablock(s) I. DOI: 10.1107/S1600536811035380/jh2321Isup2.hkl

Additional supplementary materials:  crystallographic information; 3D view; checkCIF report

## Figures and Tables

**Fig. 1.**
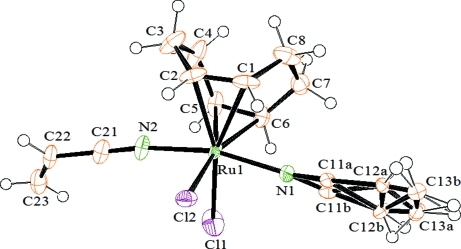
The structure of the title compound, showing 50% probability displacement ellipsoids. For the C atoms, the first digit indicates ring number and the second digit indicates the position of the atom in the ring. Some lables have been omitted for clarity.

## supplementary crystallographic information

### Comment

The present ruthenium complex, Fig.1, has been synthesized in a similar way as done earlier for the acetonitrile derivative (Chiririwa *et al.* 2011). Organonitrile solvate complexes are widely useful for synthesis of organometallic compounds because of facile substitution at the solvate coordination sites. Similarly, 1,5-cyclooctadiene complexes have found considerable use in organometallic chemistry as well.

The two acrylonitrile ligands are not *trans* to each other, as the N(2)—Ru—N(1) angle is 164.62 (11)° whereas the same angle is 163.15 (6)° in the acetonitrile derivative. This is attributed to repulsion by the alkene bonds of the COD ligand. One of the acrylonitrile ligands is slightly bent as we observed earlier in the acetonitrile derivative. The N(2)—C(21)—C(22) bond angle is 179.2 (3)°. This is probably due to packing forces.

It turned out that in the crystal structure the disorder involves three carbon atoms between the 3 positions with site occupation factors of 84:16. A l l alternative positions refined quite well without any kind of restraints and the C atoms assume positions that make an almost symmetrical system.

### Experimental

A suspension of [{RuCl_2_(COD)}*_x_*] (0.5 g) in acrylonitrile (25 ml) was refluxed for 12 h. The orange solution was filtered hot and concentrated on a steam bath to half volume and cooled to 0 °C overnight affording orange crystals in 50% yield suitable for X-ray diffraction studies.

### Refinement

The methylene, and methyl H atoms were placed in geometrically idealized positions (C—H = 0.95–0.98) and constrained to ride on their parent atoms with *U*_iso_(H) = 1.2*U*_eq_(C) for methylene H atoms, and *U*_iso_(H) = 1.5*U*_eq_(C) for methyl H atoms respectively. The acrylonitrile ligand is disordered over 3 well resolved positions. The disorder involves three C atoms which assume positions that make an almost symmetrical system. Unfortunately this disorder could not be resolved.

### Crystal data

**Table:**

[RuCl_2_(C_8_H_12_)(C_3_H_3_N)_2_]	*F*(000) = 776
*M**_r_* = 386.27	*D*_x_ = 1.684 Mg m^−^^3^
Monoclinic, *P*2_1_/*c*	Mo *K*α radiation, λ = 0.71073 Å
Hall symbol: -P 2ybc	Cell parameters from 3596 reflections
*a* = 7.1079 (8) Å	θ = 2.7–28.2°
*b* = 26.818 (3) Å	µ = 1.37 mm^−^^1^
*c* = 8.1555 (10) Å	*T* = 100 K
β = 101.408 (2)°	Rectangular, orange
*V* = 1523.9 (3) Å^3^	0.22 × 0.09 × 0.04 mm
*Z* = 4	

### Data collection

**Table:**

Bruker APEXII CCD diffractometer	3093 reflections with *I* > 2σ(*I*)
Radiation source: fine-focus sealed tube	*R*_int_ = 0.040
graphite	θ_max_ = 28.3°, θ_min_ = 1.5°
φ and ω scans	*h* = −9→8
12653 measured reflections	*k* = −35→35
3776 independent reflections	*l* = −10→10

### Refinement

**Table:**

Refinement on *F*^2^	Primary atom site location: structure-invariant direct methods
Least-squares matrix: full	Secondary atom site location: difference Fourier map
*R*[*F*^2^ > 2σ(*F*^2^)] = 0.033	Hydrogen site location: inferred from neighbouring sites
*wR*(*F*^2^) = 0.075	H-atom parameters constrained
*S* = 1.03	*w* = 1/[σ^2^(*F*_o_^2^) + (0.0254*P*)^2^ + 2.2348*P*] where *P* = (*F*_o_^2^ + 2*F*_c_^2^)/3
3776 reflections	(Δ/σ)_max_ = 0.001
200 parameters	Δρ_max_ = 1.14 e Å^−^^3^
0 restraints	Δρ_min_ = −1.11 e Å^−^^3^

### Special details

**Table:**

Geometry. All e.s.d.'s (except the e.s.d. in the dihedral angle between two l.s. planes) are estimated using the full covariance matrix. The cell e.s.d.'s are taken into account individually in the estimation of e.s.d.'s in distances, angles and torsion angles; correlations between e.s.d.'s in cell parameters are only used when they are defined by crystal symmetry. An approximate (isotropic) treatment of cell e.s.d.'s is used for estimating e.s.d.'s involving l.s. planes.
Refinement. Refinement of *F*^2^ against ALL reflections. The weighted *R*-factor *wR* and goodness of fit *S* are based on *F*^2^, conventional *R*-factors *R* are based on *F*, with *F* set to zero for negative *F*^2^. The threshold expression of *F*^2^ > σ(*F*^2^) is used only for calculating *R*-factors(gt) *etc*. and is not relevant to the choice of reflections for refinement. *R*-factors based on *F*^2^ are statistically about twice as large as those based on *F*, and *R*- factors based on ALL data will be even larger.The Following Model and Quality ALERTS were generated -(Acta-Mode) <<< Format: alert-number_ALERT_alert-type_alert-level text 912_ALERT_4_C Missing # of FCF Reflections Above STh/*L*= 0.600 7 Noted.

### Fractional atomic coordinates and isotropic or equivalent isotropic displacement parameters (Å^2^)

**Table:**

	*x*	*y*	*z*	*U*_iso_*/*U*_eq_	Occ. (<1)
Ru1	0.50503 (3)	0.634032 (9)	0.06561 (3)	0.01778 (7)	
Cl1	0.75368 (12)	0.59883 (4)	0.27841 (13)	0.0487 (3)	
Cl2	0.39293 (11)	0.68818 (3)	0.26070 (9)	0.02385 (16)	
C1	0.3571 (5)	0.67769 (12)	−0.1549 (4)	0.0284 (7)	
H1	0.3868	0.7064	−0.0866	0.034*	
C2	0.2289 (4)	0.64408 (13)	−0.1127 (4)	0.0286 (7)	
H2	0.1739	0.6513	−0.0182	0.034*	
C3	0.1699 (5)	0.59630 (15)	−0.2071 (5)	0.0404 (9)	
H3A	0.088	0.6047	−0.3165	0.048*	
H3B	0.0917	0.5762	−0.1436	0.048*	
C4	0.3393 (5)	0.56453 (14)	−0.2372 (5)	0.0403 (9)	
H4A	0.3016	0.529	−0.2387	0.048*	
H4B	0.3651	0.5727	−0.3491	0.048*	
C5	0.5230 (4)	0.57127 (12)	−0.1091 (5)	0.0283 (7)	
H5	0.5447	0.5507	−0.0124	0.034*	
C6	0.6595 (4)	0.60595 (13)	−0.1271 (4)	0.0273 (7)	
H6	0.7733	0.6072	−0.0434	0.033*	
C7	0.6410 (5)	0.64203 (13)	−0.2703 (4)	0.0333 (8)	
H7A	0.6502	0.6233	−0.373	0.04*	
H7B	0.7503	0.6656	−0.2476	0.04*	
C8	0.4539 (6)	0.67198 (14)	−0.3029 (4)	0.0369 (9)	
H8A	0.4812	0.7057	−0.3421	0.044*	
H8B	0.3627	0.6557	−0.3947	0.044*	
C23	0.2232 (5)	0.48525 (13)	0.3860 (4)	0.0343 (8)	
H23A	0.3572	0.4795	0.3995	0.041*	
H23B	0.1468	0.4638	0.4388	0.041*	
N1	0.6921 (4)	0.68978 (11)	0.0491 (3)	0.0305 (7)	
C11A	0.7543 (16)	0.7291 (4)	0.0267 (11)	0.0137 (18)	0.419 (13)
C12A	0.8507 (12)	0.7726 (3)	−0.0172 (8)	0.015 (2)	0.419 (13)
H12A	0.7749	0.8	−0.0659	0.018*	0.419 (13)
C13A	1.0368 (13)	0.7766 (3)	0.0062 (10)	0.022 (2)	0.419 (13)
H13A	1.116	0.7498	0.0546	0.027*	0.419 (13)
H13B	1.0932	0.8063	−0.0253	0.027*	0.419 (13)
C11B	0.8206 (11)	0.7163 (3)	0.0505 (9)	0.0170 (14)	0.581 (13)
C12B	0.9662 (8)	0.7536 (2)	0.0446 (6)	0.0183 (17)	0.581 (13)
H12B	1.0946	0.7477	0.1006	0.022*	0.581 (13)
C13B	0.9232 (10)	0.7954 (3)	−0.0371 (7)	0.0249 (18)	0.581 (13)
H13C	0.7951	0.8016	−0.0934	0.03*	0.581 (13)
H13D	1.0202	0.8196	−0.04	0.03*	0.581 (13)
N2	0.3376 (4)	0.58267 (10)	0.1480 (4)	0.0267 (6)	
C21	0.2527 (5)	0.55592 (12)	0.2127 (4)	0.0277 (7)	
C22	0.1436 (5)	0.52268 (13)	0.2946 (4)	0.0288 (7)	
H22	0.0094	0.5279	0.2824	0.035*	

### Atomic displacement parameters (Å^2^)

**Table:**

	*U*^11^	*U*^22^	*U*^33^	*U*^12^	*U*^13^	*U*^23^
Ru1	0.01229 (11)	0.01244 (12)	0.02730 (13)	−0.00135 (9)	0.00074 (8)	0.00569 (10)
Cl1	0.0245 (4)	0.0659 (7)	0.0537 (6)	0.0169 (4)	0.0029 (4)	0.0357 (5)
Cl2	0.0281 (4)	0.0196 (4)	0.0208 (3)	−0.0016 (3)	−0.0026 (3)	0.0012 (3)
C1	0.0391 (18)	0.0215 (16)	0.0185 (14)	0.0115 (14)	−0.0093 (13)	−0.0031 (12)
C2	0.0191 (14)	0.0326 (19)	0.0292 (16)	0.0094 (13)	−0.0074 (12)	−0.0133 (14)
C3	0.0209 (16)	0.048 (2)	0.051 (2)	−0.0056 (16)	0.0036 (15)	−0.0299 (19)
C4	0.0284 (17)	0.032 (2)	0.066 (2)	−0.0121 (16)	0.0226 (17)	−0.0284 (19)
C5	0.0253 (16)	0.0136 (15)	0.052 (2)	0.0035 (13)	0.0211 (14)	−0.0012 (14)
C6	0.0191 (14)	0.0271 (18)	0.0379 (17)	−0.0002 (13)	0.0110 (13)	0.0002 (14)
C7	0.042 (2)	0.0281 (19)	0.0309 (17)	−0.0093 (16)	0.0109 (15)	−0.0038 (14)
C8	0.058 (2)	0.030 (2)	0.0201 (15)	0.0067 (18)	0.0004 (15)	−0.0025 (14)
C23	0.039 (2)	0.0249 (18)	0.045 (2)	−0.0067 (16)	0.0227 (16)	−0.0051 (16)
N1	0.0380 (16)	0.0328 (17)	0.0196 (12)	−0.0209 (14)	0.0030 (11)	0.0011 (12)
C11A	0.016 (5)	0.009 (4)	0.015 (4)	0.004 (3)	−0.001 (3)	0.004 (3)
C12A	0.019 (4)	0.011 (4)	0.014 (3)	0.000 (3)	0.004 (3)	0.000 (3)
C13A	0.020 (5)	0.019 (4)	0.026 (4)	−0.005 (4)	−0.001 (3)	0.003 (3)
C11B	0.017 (3)	0.016 (4)	0.017 (3)	0.007 (3)	−0.001 (2)	0.000 (2)
C12B	0.014 (3)	0.018 (3)	0.021 (3)	−0.002 (2)	−0.001 (2)	−0.002 (2)
C13B	0.023 (3)	0.029 (4)	0.021 (3)	−0.005 (3)	0.001 (2)	0.001 (3)
N2	0.0216 (13)	0.0173 (14)	0.0450 (16)	−0.0011 (11)	0.0155 (12)	−0.0004 (12)
C21	0.0246 (16)	0.0177 (16)	0.0448 (19)	−0.0023 (13)	0.0163 (14)	−0.0074 (14)
C22	0.0235 (16)	0.0261 (18)	0.0420 (18)	−0.0075 (14)	0.0191 (14)	−0.0071 (15)

### Geometric parameters (Å, °)

**Table:**

Ru1—N2	2.021 (3)	C7—C8	1.532 (5)
Ru1—N1	2.023 (3)	C7—H7A	0.99
Ru1—C2	2.216 (3)	C7—H7B	0.99
Ru1—C6	2.219 (3)	C8—H8A	0.99
Ru1—C5	2.225 (3)	C8—H8B	0.99
Ru1—C1	2.228 (3)	C23—C22	1.310 (5)
Ru1—Cl2	2.4035 (8)	C23—H23A	0.95
Ru1—Cl1	2.4111 (9)	C23—H23B	0.95
C1—C2	1.373 (5)	N1—C11B	1.156 (7)
C1—C8	1.511 (5)	N1—C11A	1.171 (9)
C1—H1	0.95	C11A—C12A	1.434 (13)
C2—C3	1.511 (5)	C12A—C13A	1.303 (14)
C2—H2	0.95	C12A—H12A	0.95
C3—C4	1.534 (5)	C13A—H13A	0.95
C3—H3A	0.99	C13A—H13B	0.95
C3—H3B	0.99	C11B—C12B	1.447 (10)
C4—C5	1.513 (5)	C12B—C13B	1.309 (11)
C4—H4A	0.99	C12B—H12B	0.95
C4—H4B	0.99	C13B—H13C	0.95
C5—C6	1.373 (4)	C13B—H13D	0.95
C5—H5	0.95	N2—C21	1.132 (4)
C6—C7	1.503 (5)	C21—C22	1.431 (4)
C6—H6	0.95	C22—H22	0.95
			
N2—Ru1—N1	164.62 (11)	C3—C4—H4B	108.6
N2—Ru1—C2	78.32 (13)	H4A—C4—H4B	107.5
N1—Ru1—C2	112.03 (13)	C6—C5—C4	122.6 (3)
N2—Ru1—C6	114.26 (11)	C6—C5—Ru1	71.78 (19)
N1—Ru1—C6	77.28 (12)	C4—C5—Ru1	112.4 (2)
C2—Ru1—C6	94.29 (12)	C6—C5—H5	118.7
N2—Ru1—C5	79.02 (11)	C4—C5—H5	118.7
N1—Ru1—C5	113.25 (12)	Ru1—C5—H5	85.9
C2—Ru1—C5	80.06 (12)	C5—C6—C7	124.3 (3)
C6—Ru1—C5	35.98 (11)	C5—C6—Ru1	72.24 (19)
N2—Ru1—C1	114.32 (13)	C7—C6—Ru1	110.7 (2)
N1—Ru1—C1	76.67 (12)	C5—C6—H6	117.8
C2—Ru1—C1	36.00 (13)	C7—C6—H6	117.8
C6—Ru1—C1	80.07 (12)	Ru1—C6—H6	87.1
C5—Ru1—C1	87.62 (12)	C6—C7—C8	114.3 (3)
N2—Ru1—Cl2	84.11 (8)	C6—C7—H7A	108.7
N1—Ru1—Cl2	84.59 (9)	C8—C7—H7A	108.7
C2—Ru1—Cl2	89.67 (9)	C6—C7—H7B	108.7
C6—Ru1—Cl2	161.63 (9)	C8—C7—H7B	108.7
C5—Ru1—Cl2	161.70 (8)	H7A—C7—H7B	107.6
C1—Ru1—Cl2	92.95 (9)	C1—C8—C7	115.6 (3)
N2—Ru1—Cl1	83.67 (8)	C1—C8—H8A	108.4
N1—Ru1—Cl1	86.52 (9)	C7—C8—H8A	108.4
C2—Ru1—Cl1	161.43 (10)	C1—C8—H8B	108.4
C6—Ru1—Cl1	88.96 (9)	C7—C8—H8B	108.4
C5—Ru1—Cl1	92.18 (10)	H8A—C8—H8B	107.4
C1—Ru1—Cl1	161.56 (10)	C22—C23—H23A	120
Cl2—Ru1—Cl1	92.97 (3)	C22—C23—H23B	120
C2—C1—C8	124.3 (3)	H23A—C23—H23B	120
C2—C1—Ru1	71.52 (18)	C11B—N1—Ru1	169.3 (5)
C8—C1—Ru1	112.0 (2)	C11A—N1—Ru1	161.6 (6)
C2—C1—H1	117.9	N1—C11A—C12A	170.1 (10)
C8—C1—H1	117.9	C13A—C12A—C11A	123.5 (9)
Ru1—C1—H1	86.4	C13A—C12A—H12A	118.3
C1—C2—C3	124.1 (3)	C11A—C12A—H12A	118.3
C1—C2—Ru1	72.48 (17)	C12A—C13A—H13A	120
C3—C2—Ru1	110.6 (2)	C12A—C13A—H13B	120
C1—C2—H2	118	H13A—C13A—H13B	120
C3—C2—H2	118	N1—C11B—C12B	173.7 (8)
Ru1—C2—H2	86.9	C13B—C12B—C11B	120.9 (7)
C2—C3—C4	113.9 (3)	C13B—C12B—H12B	119.6
C2—C3—H3A	108.8	C11B—C12B—H12B	119.6
C4—C3—H3A	108.8	C12B—C13B—H13C	120
C2—C3—H3B	108.8	C12B—C13B—H13D	120
C4—C3—H3B	108.8	H13C—C13B—H13D	120
H3A—C3—H3B	107.7	C21—N2—Ru1	171.8 (3)
C5—C4—C3	114.9 (3)	N2—C21—C22	179.2 (3)
C5—C4—H4A	108.6	C23—C22—C21	122.0 (3)
C3—C4—H4A	108.6	C23—C22—H22	119
C5—C4—H4B	108.6	C21—C22—H22	119
			
N2—Ru1—C1—C2	−0.5 (2)	N1—Ru1—C5—C4	−116.6 (2)
N1—Ru1—C1—C2	−169.1 (2)	C2—Ru1—C5—C4	−6.8 (3)
C6—Ru1—C1—C2	111.7 (2)	C6—Ru1—C5—C4	−118.5 (4)
C5—Ru1—C1—C2	76.3 (2)	C1—Ru1—C5—C4	−42.2 (3)
Cl2—Ru1—C1—C2	−85.38 (18)	Cl2—Ru1—C5—C4	49.9 (5)
Cl1—Ru1—C1—C2	166.0 (2)	Cl1—Ru1—C5—C4	156.2 (2)
N2—Ru1—C1—C8	−120.9 (2)	C4—C5—C6—C7	2.2 (5)
N1—Ru1—C1—C8	70.5 (3)	Ru1—C5—C6—C7	−103.3 (3)
C2—Ru1—C1—C8	−120.4 (3)	C4—C5—C6—Ru1	105.5 (3)
C6—Ru1—C1—C8	−8.7 (2)	N2—Ru1—C6—C5	12.6 (2)
C5—Ru1—C1—C8	−44.1 (3)	N1—Ru1—C6—C5	−178.2 (2)
Cl2—Ru1—C1—C8	154.2 (2)	C2—Ru1—C6—C5	−66.5 (2)
Cl1—Ru1—C1—C8	45.7 (4)	C1—Ru1—C6—C5	−99.7 (2)
C8—C1—C2—C3	1.1 (5)	Cl2—Ru1—C6—C5	−168.5 (2)
Ru1—C1—C2—C3	−103.4 (3)	Cl1—Ru1—C6—C5	95.1 (2)
C8—C1—C2—Ru1	104.5 (3)	N2—Ru1—C6—C7	133.4 (2)
N2—Ru1—C2—C1	179.5 (2)	N1—Ru1—C6—C7	−57.4 (2)
N1—Ru1—C2—C1	11.4 (2)	C2—Ru1—C6—C7	54.3 (2)
C6—Ru1—C2—C1	−66.6 (2)	C5—Ru1—C6—C7	120.8 (3)
C5—Ru1—C2—C1	−99.8 (2)	C1—Ru1—C6—C7	21.1 (2)
Cl2—Ru1—C2—C1	95.47 (18)	Cl2—Ru1—C6—C7	−47.7 (4)
Cl1—Ru1—C2—C1	−166.1 (2)	Cl1—Ru1—C6—C7	−144.0 (2)
N2—Ru1—C2—C3	−59.9 (3)	C5—C6—C7—C8	51.7 (5)
N1—Ru1—C2—C3	132.0 (3)	Ru1—C6—C7—C8	−30.5 (3)
C6—Ru1—C2—C3	54.0 (3)	C2—C1—C8—C7	−87.3 (4)
C5—Ru1—C2—C3	20.8 (3)	Ru1—C1—C8—C7	−5.3 (4)
C1—Ru1—C2—C3	120.6 (3)	C6—C7—C8—C1	24.1 (4)
Cl2—Ru1—C2—C3	−144.0 (3)	N2—Ru1—N1—C11B	72 (2)
Cl1—Ru1—C2—C3	−45.6 (4)	C2—Ru1—N1—C11B	−157 (2)
C1—C2—C3—C4	50.4 (5)	C6—Ru1—N1—C11B	−68 (2)
Ru1—C2—C3—C4	−31.9 (4)	C5—Ru1—N1—C11B	−69 (2)
C2—C3—C4—C5	27.1 (5)	C1—Ru1—N1—C11B	−150 (2)
C3—C4—C5—C6	−90.6 (4)	Cl2—Ru1—N1—C11B	115 (2)
C3—C4—C5—Ru1	−8.5 (4)	Cl1—Ru1—N1—C11B	22 (2)
N2—Ru1—C5—C6	−168.3 (2)	N2—Ru1—N1—C11A	−101.0 (15)
N1—Ru1—C5—C6	1.9 (2)	C2—Ru1—N1—C11A	29.3 (15)
C2—Ru1—C5—C6	111.8 (2)	C6—Ru1—N1—C11A	118.8 (15)
C1—Ru1—C5—C6	76.3 (2)	C5—Ru1—N1—C11A	117.7 (15)
Cl2—Ru1—C5—C6	168.5 (2)	C1—Ru1—N1—C11A	36.2 (15)
Cl1—Ru1—C5—C6	−85.2 (2)	Cl2—Ru1—N1—C11A	−58.1 (15)
N2—Ru1—C5—C4	73.1 (3)	Cl1—Ru1—N1—C11A	−151.4 (15)
